# Efficient Synthesis of Ethanol from CH_4_ and Syngas on a Cu-Co/TiO_2_ Catalyst Using a Stepwise Reactor

**DOI:** 10.1038/srep34670

**Published:** 2016-10-03

**Authors:** Zhi-Jun Zuo, Fen Peng, Wei Huang

**Affiliations:** 1 Key Laboratory of Coal Science and Technology of Ministry of Education and Shanxi Province, Taiyuan University of Technology, Taiyuan 030024, Shanxi, China; 2 Key Laboratory of Renewable Energy and Gas Hydrate, Guangzhou Institute of Energy Conversion, Chinese Academy of Sciences, Guangzhou, China

## Abstract

Ethanol synthesis from CH_4_ and syngas on a Cu-Co/TiO_2_ catalyst is studied using experiments, density functional theory (DFT) and microkinetic modelling. The experimental results indicate that the active sites of ethanol synthesis from CH_4_ and syngas are Cu and CoO, over which the ethanol selectivity is approximately 98.30% in a continuous stepwise reactor. DFT and microkinetic modelling results show that *CH_3_ is the most abundant species and can be formed from *CH_4_ dehydrogenation or through the process of *CO hydrogenation. Next, the insertion of *CO into *CH_3_ forms *CH_3_CO. Finally, ethanol is formed through *CH_3_CO and *CH_3_COH hydrogenation. According to our results, small particles of metallic Cu and CoO as well as a strongly synergistic effect between metallic Cu and CoO are beneficial for ethanol synthesis from CH_4_ and syngas on a Cu-Co/TiO_2_ catalyst.

Owing to the diminishing supply of fossil fuels and rising crude oil prices, an alternative fuel source must be developed. Ethanol synthesis has recently attracted increasing attention because of its nontoxic nature and ability to be produced from renewable sources^1^. In general, there are two main methods of ethanol synthesis: one is fermentation derived from corn or sugar cane and hydration of petroleum-based ethylene, and the other is CO hydrogenation^1^,^2^,^3^,^4^,^5^,^6^. Ethanol synthesis from syngas has recently received attention owing to food shortages. To the best of our knowledge, Rh-based catalysts are the best catalysts that show relatively high ethanol selectivity^7^,^8^,^9^,^10^. However, the high cost of Rh limits its application in industry.

C_2_-oxygenate synthesis from CH_4_ and CO_2_ is thermodynamically unfavourable at low temperatures, but this can be overcome through a stepwise reaction technology that has been proposed by our group^11^. In this process, *CH_4_ is first adsorbed on the catalyst surface (M) and then dissociated to generate CH_x_-M; subsequently, the *CO_2_ species is inserted into the C-M bond to form *CH_x_COO before finally forming acetic acid from *CH_x_COO hydrogenation^12^,^13^,^14^. It was found that the Pd-Co and Cu-Co bi-metal supported on TiO_2_ catalysts exhibited good activity for acetic acid from CH_4_ and CO_2_^15^. Because CO_2_ has a relatively high reduction potential (1.9 V to CO_2_^−^), the conversion is difficult^16^. If CO_2_ is replaced by CO, then the conversion of CO is possibly better than that of CO_2_. Therefore, we propose a method of ethanol synthesis from CH_4_ and syngas in a stepwise reactor.

Although the activity of the Pd-Co/TiO_2_ catalysts is better than that of the Cu-Co/TiO_2_ catalysts for acetic acid synthesis from CH_4_-CO_2_ in the stepwise reactor^15^, we chose the Cu-Co/TiO_2_ catalysts for ethanol synthesis from CH_4_ and syngas, considering the price of Pd. Finally, the reaction mechanisms of ethanol from CH_4_ and syngas were studied on Cu-Co/TiO_2_ using density functional theory (DFT) and microkinetic modelling. The result may be useful for computational design and optimizations of Cu-Co/TiO_2_ catalysts.

## Result and Discussion

### Experimental result

**Figure 1:**
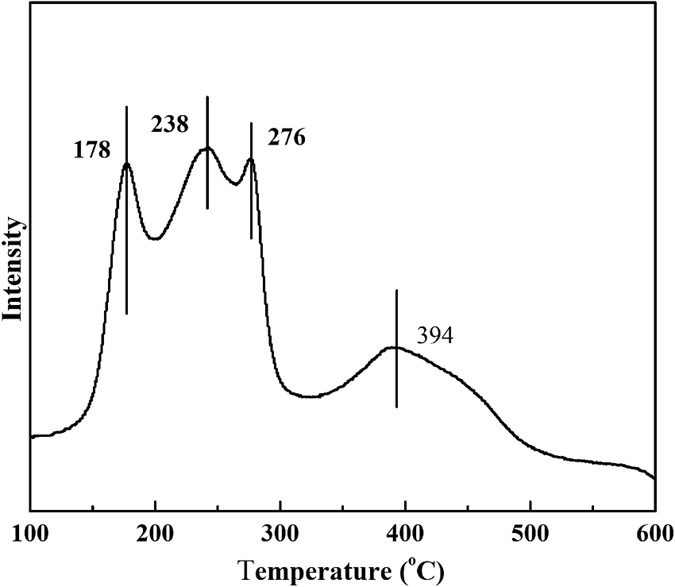
H_2_-TPR profile before reaction.

Figure 1 shows the H_2_-TPR profile of the Cu-Co/TiO_2_ catalyst before reaction. The H_2_-TPR curves show four main peaks. The peak at 178 °C can be assigned as the reduction of CuO to Cu, and the peak at 238 °C is attributed to Cu–Co spinal phase (such as Cu_x_Co_3-x_O_4_ oxides)^4^,^17^. The peaks at approximately 276 and 394 °C are assigned the reduction of Co_3_O_4_ → CoO and CoO → Co^18^,^19^,^20^. Note that the reduction temperatures of Co_3_O_4_ → CoO and CoO → Co are approximately 450 and 550 °C^18^, which are higher than that of our catalyst. The reason for this observation is that the Cu species are first reduced at low temperatures to form metallic Cu nanoparticles, which subsequently catalyse the reduction of nearby Co species^18^,^19^. As a result, the reduction temperature of the Co species in the Cu-Co/TiO_2_ catalyst is lower than that of the pure Co species. No reduction peak of TiO_2_ is detected, which is in accordance with our X-ray powder diffraction (XRD) (Fig. S1) results. XRD and high-resolution transmission electron microscopy (TEM) (Fig. S2) also show that Cu species and Co species are uniformly dispersed on the catalyst surface.

**Figure 2:**
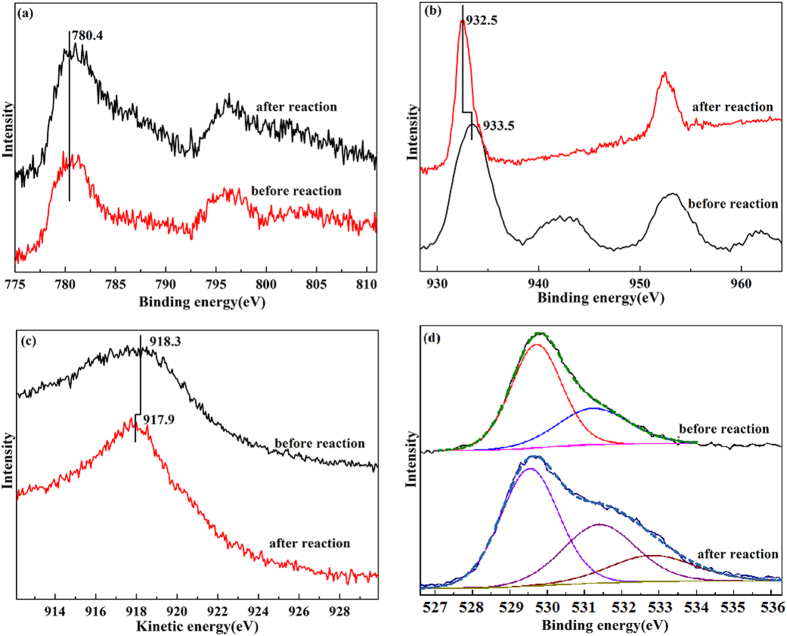
Co 2p (**a**), Cu 2p (**b**), Cu LMM (**c**) and O 1s (**d**) XPS spectra before and after reaction.

Figure 2 displays the Co 2p, Cu 2p, Cu LMM, and O 1s XPS spectra of the Co-Cu/TiO_2_ catalyst before and after reaction. As shown in Fig. 2a, the binding energies of Co 2p3 before and after are similar to each other, being located at approximately 780.4 eV. The intensity of the shakeup satellite of Co 2p3 before the reaction is obviously lower than that after the reaction. Therefore, the Co species before the reaction is mainly Co_3_O_4_ and Cu–Co spinal phase, a similar shape of the Co 2p3/2 core level spectra is also observed for mixed Cu_x_Co_3-x_O_4_ oxides^21^,^22^,^23^. After reaction, CoO is the main phase^21^,^24^. This result is similar to our previous result, in which CoO is the main phase in the Co-Pd/TiO_2_ catalysts under 400 °C for a 2 h reduction using *in-situ* XPS^21^. Note that some CoO is reduced to metallic Co at 400 °C according to the TPR result, but the metallic Co is not detected by XPS. The reason for this observation is that a small amount of CoO is reduced at approximately 400 °C, i.e., much CoO is not reduced, according to the TPR result. Thus, the Co 2p3 peak of CoO overlaps with that of metallic Co, and the intensity of CoO is larger than that of metallic Co; as a result, the metallic Co is not detected by XPS.

For Cu 2p (Fig. 2b), a shakeup satellite is observed at approximately 942 eV before the reaction, and the binding energy of Cu 2p3 before the reaction is approximately 933.5 eV, which can be assigned to Cu^2+^ (CuO, 933.7 eV)^25^,^26^. The result shows that the surface is covered by CuO before the reaction. After the reaction, the shakeup satellite disappears, indicating that the CuO is reduced. The metallic Cu and Cu_2_O cannot be distinguished using Cu 2p3, whereas they could be distinguished from the Cu LMM Auger spectra (Fig. 2c). As shown in Fig. 2c, the kinetic energy of Cu LMM after the reaction is approximately 917.9 eV. The kinetic energy is slightly smaller than the kinetic energy of metallic Cu (918.4 eV) but is obviously larger than that of Cu_2_O (916.2 eV)^25^,^26^.This result indicates that the surface is covered by metallic Cu after the reaction. The kinetic energy of Cu LMM is approximately 918.3 eV, which is similar to the kinetic energy of CuO (918.1 eV)^25^,^26^, further verifying the presence of CuO on the surface before the reaction.

In the case of O 1s (Fig. 2d), the peaks at 529.7 and 531.3 eV are assigned as lattice oxygen and O(H) species respectively before reaction^27^. After reaction, a new peak appears at 532.7 eV. The peak can be attributed to C=O or O-C-O^28^, because the productions adsorb on the surface. For the Ti species, the binding energies are approximately 458.5 eV before and after the reaction (Fig. S3), which can be assigned to the TiO_2_^29^. The result shows that the TiO_2_ could not be reduced during the reaction. In general, the surface is mainly covered by CuO and Co_3_O_4_ before the reaction, whereas the surface is mainly covered by Cu and CoO after the reaction, in agreement with the H_2_-TPR result. In other words, the metallic Cu and CoO are the active sites for the CH_4_-syngas conversion.

**Figure 3:**
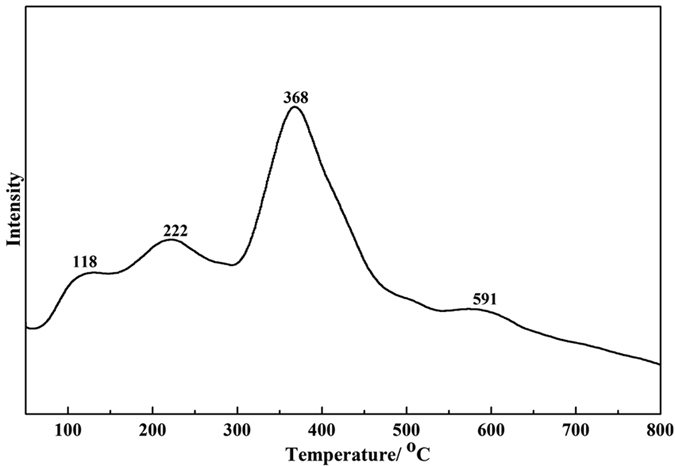
NH_3_-TPD before reaction.

Figure 3 shows the NH_3_-TPD spectra of the Cu/TiO_2_ catalyst. A larger peak and four main NH_3_ desorption peaks are detected. The first peak at approximately 118 °C is attributed to the weak acid, the second and third peaks are assigned to the mediate strong acid, and the peak at 591 °C is assigned to the strong acid. Our group has been studying the activation and conversion of CO_2_ and CH_4_ over Cu-Co catalysts supported on different solid acid supports, such as γ-Al_2_O_3_, ZrO_2_/SO_4_^2−^, and HZSM5^30^. The activation ability of CH_4_ on the Cu-Co catalyst increases with increasing acid intensity, but the too strong acid is not beneficial for the formation of active species. In other words, the appropriate acid intensity of the Co-Cu/TiO_2_ catalyst is favours for the conversion of CH_4_ and syngas.

Table 1 shows the formation rate and the selectivity of the products on the Cu-Co/TiO_2_ catalyst. As shown in Table 1, the formation rates of CH_3_OH, C_2_H_5_OH and CH_3_COOH are 1.90, 139.37 and 0.51 mg · g_cat._^−1^·h^−1^, respectively, and the corresponding selectivities of CH_3_OH, C_2_H_5_OH and CH_3_COOH are 1.34%, 98.30% and 0.36%, respectively. The result shows that the formation rate and selectivity of C_2_H_5_OH are far greater than those of CH_3_OH and CH_3_COOH, indicating that the Cu-Co/TiO_2_ catalyst is beneficial for the formation of C_2_H_5_OH. In addition, only C_2_H_5_OH, CH_3_OH, and CH_3_COOH are produced; these species are easily separated.

### DFT results

#### Ethanol synthesis from CH_4_ and syngas on CoCu(111) surface

The adsorption configurations of possible intermediates involved ethanol synthesis from CH_4_ and syngas on the CoCu(111) surface are shown in Fig. S4, and the corresponding adsorption parameters are listed in Table 2. Figures S5–S10 show the energy barriers, the reaction energies and the TS structures of ethanol synthesis from CH_4_ and syngas on the CoCu(111) surface.

As shown in Figs S5 and S6, *CH_4_ dehydrogenation are as follows: *CH_4_ → *CH_3_ → *CH_2_→ *CH → *C, which is in accordance with the previous studies of CH_4_ dehydrogenation on different metals and alloys using DFT^31^,^32^,^33^,^34^,^35^,^36^,^37^,^38^,^39^. Table S1 shows the adsorption parameters of *CH_4_, *CH_3_, *CH_2_, *CH and *C on a Cu site. Comparing Table 2 with Table S1, the binding strengths of *CH_3_, *CH_2_, *CH and *C on a Co site (−1.69, −4.23, −5.81 and −6.43 eV) are found to be obviously larger than those on a Cu site (−1.23, −3.82, −5.21 and −5.46 eV); the binding strength of *H on a Co site (−2.67 eV) is slightly larger than that on a Cu site (−2.45 eV); and the binding strength of *CH_4_ on a Co site (−0.11 eV) is similar to that on a Cu site (−0.10 eV). The observed trend is in agreement with the result of Liu *et al.*^37^. We also studied *CH_3_ and *CH_2_ formation on fcc_Cu_ (Fig. S7); the energy barriers are 1.92 and 1.18 eV, which are far larger than that on a Co site. The result indicates that CH_4_ dehydrogenation prefers to occur on Co sites versus Cu sites.

As shown in Fig. S8, *CHO and *CH_2_O formation are likely from *CO hydrogenation. Again, *CH_2_O hydrogenation is superior to dissociation. Because the energy barrier of *CH_3_O formation (0.89 eV) is similar to that of *CH_2_OH formation (0.82 eV), *CH_2_OH and *CH_3_O further reactions are considered. In the case of *CH_2_OH further reaction, *CH_2_ formation occurs slightly easier than *CH_3_OH formation. Similarly, *CH_3_O prefers to be dissociated into *CH_3_ and *O. A previous study showed that the energy barrier of *CH_3_O+ *H → *CH_3_OH + * (0.76 eV) is lower than that of *CH_3_O+ * → *CH_3_ + *O (1.05 eV) on a Rh(111) surface^40^. The energy barrier of *CH_3_O+ *H → *CH_3_OH +* (1.07 eV) is obviously smaller than that of *CH_3_O+ * → *CH_3_ + *O (2.22 eV) on a Cu(211) surface; however, the energy barrier of *CH_3_O+ *H → *CH_3_OH +* (1.41 eV) is slightly lower than that of *CH_3_O+ * → *CH_3_ + *O (1.67 eV) on a Rh doped Cu(211) surface. Thus, Zhang *et al.* considered that C-O scission is difficult to perform on Cu-based catalysts and the promoter Rh facilitates *CH_3_ formation. The results show that the promoter Rh increases the productivity and selectivity of ethanol synthesis from syngas on Cu-based catalysts^41^. The energy barrier and reaction energy of *CH_3_OH+ * → *CH_3_ + *OH are 0.81 and 0.10 eV, and the energy barrier of *CH_3_OH+ * → *CH_3_ + *OH is higher than that of the desorption energy of CH_3_OH (0.51 eV). This result shows that CH_3_OH desorption occurs on the surface.

According to the above results, *CH_3_, *CH_2_, *CH and *C are the possible intermediates during the process of *CH_4_ dehydrogenation, and *CH_3_ and *CH_2_ are the possible intermediates from C-O scission during the process of methanol from syngas. Therefore, *CH_3_, *CH_2_, *CH and *C reactions with *CO are considered in this section. As shown in Fig. S9, the energy barriers of *CH_3_CO, *CH_2_CO, *CHCO and *CCO are in the following order: *CH_3_CO (0.49 eV) > *CH_2_CO (1.55 eV) > *CHCO (1.71 eV) > *CCO (2.07 eV). The result shows that the insertion ability of *CO decreases with decreasing H number of *CH_x_(x = 0, 1, 2, 3). Finally, *C_2_H_5_OH is synthesized through *CH_3_COH and *CH_3_CHOH from *CH_3_CO further hydrogenation (Fig. S10).

It is well known that the Cu-based catalysts also have applications in the water-gas shift (WGS) reaction or the reverse WGS reaction^42^,^43^,^44^. Because of the complication of both reactions, we only consider *CO_2_ and *H_2_O formation. The energy barriers of *CO + *O → *CO_2_ + * and *OH + *H → *H_2_O + * are 0.81 and 1.43 eV, respectively (Fig. S10). The energy barrier of *CH_3_+ *CO_2_ → *CH_3_COO + * are 1.13 eV, which is higher than those of *CH_3_CO (0.49 eV) and *C_2_H_6_ (0.89 eV) formation, indicating that CH_3_COO formation is difficult. There is only one product of *CH_3_COO hydrogenation, *CH_3_COOH, for which the energy barrier and reaction energy are 0.93 and 0.08 eV, respectively.

#### Ethanol synthesis from CH_4_ and syngas on Cu(111) and Co(111) surfaces

According to the above result, there are two key factors for ethanol synthesis: one is *CH_x_ formation; the other is C-C bond formation. For *CH_x_ formation, there are two methods: one is from CH_4_ decomposition; the other is from C-O bond scission during the process of methanol synthesis. Liu *et al.* studied CH_4_ decomposition on Co(111) and Cu(111) surfaces^37^. They found that the energy barrier of *CH_3_ formation from *CH_4_ dehydrogenation on a Cu(111) surface is obviously higher than that on a Co(111) surface (1.14 vs. 1.88 eV) using the same calculation parameters. The results show that CH_4_ decomposition preferably occurs on a Co site.

Regarding *CH_x_ formation during the process of methanol synthesis from syngas, our previous results showed that the energy barriers of *CH_3_OH and *CH_3_ formation from *CH_3_O, *CH_3_O and *CH_2_ formation from *CH_2_O, and *CH_2_O and *CH formation from *CHO on a Cu(111) surface are 0.63 and 2.18 eV, 1.00 and 2.05 eV, and 0.93 and 2.05 eV, respectively^45^. Zhang *et al.* and Mehmood *et al.* studied ethanol synthesis from a Cu(211) surface and methanol decomposition on Cu_4_ nanoparticles; they also found that the ability of hydrogenation is greater than that of C-O scission^41^,^46^. The result indicates that *CH_x_ is not formed on a Cu(hkl) surface during methanol synthesis. In the case of a Co surface, Mehmood *et al.* proposed that the energy barriers of *CH_3_OH and *CH_3_ formation from *CH_3_O, *CH_3_O and *CH_2_ formation from *CH_2_O, and *CH_2_O and *CH formation from *CHO on Co_4_ nanoparticle are 1.48 and 1.66 eV, 0.86 and 1.11 eV, and 1.43 and 2.13 eV, respectively^46^. The energy barriers of *CH_3_OH and *CH_3_ formation from *CH_3_O and *CH_3_O and *CH_2_ formation from *CH_2_O are similar to each other, but the energy barrier of *CH formation is higher than that of *CH_2_O formation. The result shows that *CH_2_ and *CH_3_ species formation are likely on a Co surface.

According to the above results, it was found that the formation of *CH, *CH_2_ and *CH_3_ during the process of *CH_4_ dehydrogenation and *CH_3_OH formation on a single Co active site are possible; however, it is impossible on a single Cu active site. Therefore, we only consider C-C formation on the Co(111) surface. The energy barriers, reaction energies and TSs are shown in Fig. S11.

Fig. S11 shows that the energy barriers of *C_2_H_6_, *CH_3_CO, *CH_2_CO and *CHCO formation are 0.76, 1.04, 1.40 and 2.02 eV, respectively. Comparing the energy barriers of C-C formation on the CoCu(111) surface, it was found that the energy barriers of *C_2_H_6_ (0.76 vs. 0.89 eV) and *CH_2_CO (1.40 vs. 1.55 eV) formation on the Co(111) surface are slightly lower than those on the CoCu(111) surface, whereas the energy barriers of *CH_3_CO (1.04 vs. 0.49 eV) and *CHCO (2.02 vs. 1.71 eV) formation on the Co(111) surface are higher than those on the CoCu(111) surface. The result also shows that Co-Cu based catalysts change the reaction path. In addition, the energy barrier of *C_2_H_6_ formation is lower than those of *CH_3_CO, *CH_2_CO and *CHCO formation. The result shows that *C_2_H_6_ formation is preferable, for which hydrocarbon formation is preferred versus C_2_ oxygenate. The result is in agreement with the experiment results in which a Co-based catalyst is one of catalysts for the F-T reaction^47^,^48^,^49^. Therefore, ethanol synthesis from CH_4_ and syngas requires two active sites: CoO and metallic Cu. In addition, because ethanol synthesis from CH_4_ and syngas requires a synergistic effect between metallic Cu and CoO, small particles of CoO and metallic Cu are required.

### Microkinetic modelling

To date, most possible reactions during the reaction of CH_4_ and syngas have been studied using DFT. Table 3 summarises the optimal reaction pathways for ethanol synthesis on the CoCu(111) surface together with the corresponding activation barriers. In this section, to estimate the results from DFT, the selectivity of the possible products involved in ethanol synthesis from CH_4_ and syngas under our experimental condition was studied using a microkinetic model^50^. Similar kinetic modelling has been successfully applied for various reactions^40^,^51^,^52^. As shown in Table 3, the adsorption processes (R1, R2 and R3) are assumed to be in equilibrium. The other surface species involved in the R4-R22 reaction can be described according to the pseudo-steady-state approximation^50^. The relative selectivity (s) values are defined as s_i_ = r_i_/_i_, where r is the relative rate for each product, and i denotes CH_3_OH, C_2_H_5_OH, C_2_H_6_, CH_3_COOH and H_2_O. The detailed description of the microkinetic model is shown in the supplement.

According to our DFT results and the microkinetic model, the relative selectivity of CH_3_OH, C_2_H_5_OH, C_2_H_6_, CH_3_COOH and H_2_O are determined under our experimental conditions (P_CH4_ = 0.95 atm, P_CO_ = 0.5 atm, =0.5 atm and T = 300 °C). As shown in Table 1, the relative selectivities of CH_3_OH and C_2_H_5_OH are 11.23 and 88.77%; the relative selectivities of C_2_H_6_, CH_3_COOH and H_2_O are very small and can be ignored. Compared with the experiment result, it is found that the selectivity of CH_3_OH using the microkinetic model is higher than that the experiment result, whereas the selectivities of C_2_H_5_OH and CH_3_COOH using the microkinetic model are lower than the experiment results. The differences in selectivity between our theoretical and experimental results could be caused by many effects. The first possible reason is that the Cu-Co alloy is not formed during 400 °C calcinations^53^,^54^, and the experiment results show that the Cu-CoO interface is the best model. The second possible explanation for the selectivity differences between our theoretical and experimental results is the presence of defect sites. To our best knowledge, defects can have a major role in catalysis by affecting the energy barriers^55^,^56^,^57^,^58^. Nonetheless, ethanol synthesis from CH_4_ and syngas on CoCu(111) provides useful insights into the experiment to a certain degree. In the future, we plan to investigate the Cu-CoO interface and defects for ethanol synthesis from CH_4_ and syngas.

## Conclusions

In the paper, ethanol synthesis from CH_4_ and syngas on a CoCu-based catalyst was studied using experiments, DFT and microkinetic modelling. The experimental results indicated that ethanol can be synthesised at high efficiency from CH_4_ and syngas on the Cu-Co/TiO_2_ catalyst, over which the selectivity of ethanol is approximately 98.30%. It was found that the active sites of ethanol synthesis are metallic Cu and CoO, with metallic Cu and CoO uniformly dispersed on the catalyst surface.

Most possible ethanol formation pathways from methanol and syngas were systematically investigated on CoCu(111) surface. The DFT result showed that ethanol synthesis from CH_4_ and syngas requires the synergistic effect between metallic Cu and CoO, and ethanol is not synthesised on single metallic Cu and CoO. On the CoCu(111) surface, *CH_3_ is the primary CH_x_ species. *CH_3_ forms via three pathways: the first is *CH_4_ dehydrogenation, the second is C-O scission of *CH_3_O, and the third is CH_2_ hydrogenation from C-O scission of *CH_2_OH. Subsequently, *CH_3_CO forms from the *CO and *CH_3_ reaction. Finally, ethanol is synthesised through *CH_3_COH hydrogenation. The microkinetic modelling result showed that there is only CH_3_OH and C_2_H_5_OH, for which the selectivity of ethanol is lower than that of the experiment result. We think that the difference between the theoretical and experimental results could be mainly caused by issues with the model and the presence of defect sites. Future work will focus on the Cu-CoO interface and defects for ethanol synthesis from CH_4_ and syngas.

### Experimental and theoretical methods

#### Catalyst preparation

The preparation of the support TiO_2_ was as follows: 24 g of NaOH was introduced into 60 mL of distilled water (10 M NaOH solution), and then, 1.0 g of commercial TiO_2_ powder (P25, Degussa) was dispersed into the 10 M NaOH solution with continuous stirring for 2 h. The mixture was transferred into a Teflon-lined autoclave, and then, the mixture was heated to 150 °C for 24 h under sealed conditions. Subsequently, the mixture was allowed to cool to room temperature. The powder was washed using distilled water until the pH of the powder was approximately 7. The neutral powder was washed using 0.1 mol/L HNO_3_ and then washed again using distilled water until the pH of the powder was approximately 7. After drying for 10 h at 75 °C, the obtained precipitate was calcined in air at 400 °C for 10 h, and the heating rate was 1 °C /min. Finally, the support TiO_2_ was obtained^59^,^60^.

The preparation method of the Cu-Co/TiO_2_ catalyst was the equal volume impregnation method. TiO_2_, Co(NO_3_)_2_·6H_2_O and Cu(NO_3_)_2_·3H_2_O were dissolved into ethylene glycol solution. After stirring for 12 h, the resulting slurry was dried for 12 h at 150 °C. Subsequently, the catalyst was calcined in air at 400 °C for 4 h at the heating rate of 2 °C/min. Finally, the Cu-Co/TiO_2_ catalyst was obtained^21^. The Cu and Co loading on TiO_2_ were 12 and 6 wt.%.

#### Catalyst characterization

XPS was performed using a V.G. Scientific ESCALAB250 with focused monochromated Al Kα. The residual pressure inside the analysis chamber was set to <2.0 × 10^−9^ mbar. For H_2_ temperature-programmed reduction (TPR) experiment, 50 mg catalyst was loaded into a fixed-bed reactor. The heating rate was 10 °C/min until the temperature is 600 °C using a temperature controller. The reduction gas was H_2_ and N_2_ which the ratio was 5:95 with a flow rate of 30 mL/min. NH_3_-TPD experiment was used on a TP-5000 instrument. 100 mg catalyst adsorbed NH_3_ at 50 °C until saturation, then purged the physisorbed NH_3_ using He for 30 min. Finally, the NH_3_-TPD data were collected in flow He from 50 to 800 °C which the heating rate was 10 °C/min.

#### Catalytic activity test

**Figure 4:**
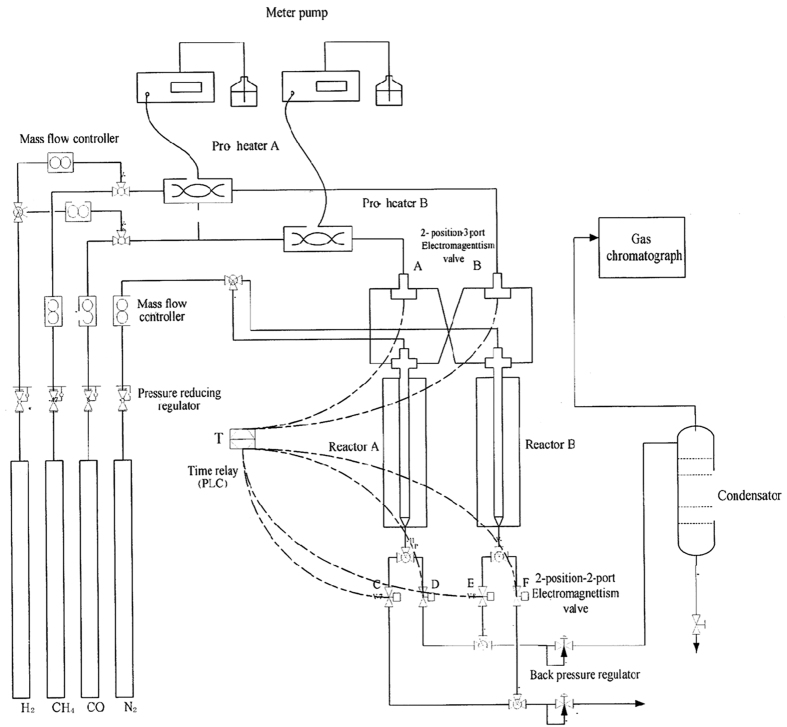
Schematic diagram of the experimental apparatus.

The diagram of the reaction apparatus is shown in Fig. 4 and was the same as the reaction apparatus of our previous paper on acetic acid synthesis from CH_4_ and CO_2_^12^. There was 1.5 g of catalysts used in reactor A and B, respectively. Before the reaction, the catalyst in both reactor A and B was reduced with 30 vol % H_2_ and 70 vol % N_2_ at 400 °C for 2 h. Because H_2_ was found to inhibit the excessive dehydrogenation of methane during CH_4_ activation, CH_4_ and H_2_ were injected together^12^. The reaction was carried out at 300 °C at atmospheric pressure. The test procedure is as follows: first, 50 ml/min of CH_4_ and 5 ml/min of H_2_ were injected into reaction A; at the same time, syngas (50 ml/min of CO and 50 ml/min of H_2_) were injected into reaction B. After 300 s, the electromagnetic valve was changed over. Then, syngas (50 ml/min of CO and 50 ml/min of H_2_) were injected into reaction A, and at the same, 50 ml/min of CH_4_ and 5 ml/min of H_2_ were injected into reaction B. Subsequently, the cycle was repeated until the reaction was finished, and ethanol was produced from CH_4_ and syngas.

The obtained products from the reaction were analysed using a gas chromatograph (GC-950) equipped with a hydrogen flame detector and packed column. The contents of each component were studied using the external standard method. On-line gas phase analysis and off-line analysis of the liquid products were performed, with the off-line analysis assisting in the product identification. The detailed analysis procedure used is as follows. The products were cooled by a condensator. Next, the liquid products were obtained from the condensator every hour and were injected into the GC. The gas phase was collected every 70 s; the gas was not cooled and condensed by the condensator. Only the oxy-organics in the gas phase were considered. The space time yield (STY, S), the number of total moles of the hydrocarbon (n), and the selectivity of carbon atoms (x) were defined as S = K × A × V/m, n = and x = S × N/(n × M), where K, A, V, m, N and M are a constant using the external standard method, the area of products (i) using chromatography, gas flow, mass of catalyst, carbon number of the products and molar mass of products, respectively.

### Computational methods

The geometries and transition state (TS) were calculated using the Dmol^3^ Materials Studio software^61^,^62^. The calculation parameters were the same as those in our previous studies^45^,^63^. The electronic structures were obtained by solving the Kohn−Sham equation self-consistently under spin-unrestricted conditions^64^,^65^. DFT was also used for the core electrons by applying the PW91 generalised-gradient approximation to the exchange-correlation energy^66^. A double numeric quality basis set with polarisation functions was used. A self-consistent field procedure is carried out with a convergence criterion of 10^−5^ a.u. on energy and electron density, and the geometry is optimized under a symmetry constraint, with the convergence criteria of 10^−3^ a.u. on the gradient and 10^−3^ a.u. on the displacement. The TS was identified using the complete linear/quadratic synchronous transit method^67^.

**Figure 5:**
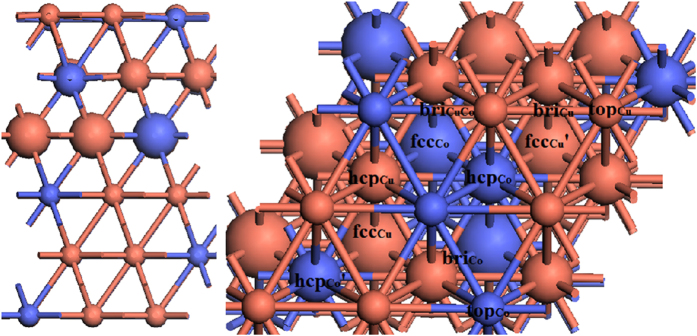
Side view (left) and top (right) view of the CoCu(111) surface after optimization.

The Cu(111) surface was cleaved from the face-centred cubic (fcc) crystal structure after optimisation; the theoretical equilibrium lattice constant of Cu was a_Cu_ = 3.685 Å, compared with the experimental value of a_Cu_ = 3.604 Å^68^. The surface was modelled using a six-layered mode p(3 × 3) super cell with nine atoms in each layer along with a 15 Å vacuum slab. The mass ratio of Cu:Co was 2 in the experiment, for which the molar ratio was approximately 1.8, and the molar ratio of Cu:Co of the CoCu(111) surface was 2 to simplify the model building. Next, three Cu atoms were replaced by Co atoms in each of the layers. The structure of the CoCu(111) surface after optimisation is shown in Fig. 5. During the calculation process, the bottom two layers were fixed, and other layers and adsorbates were allowed to relax. Meshes of 3 × 3 × 1 k-points were used for the CoCu(111) and Co(111) surfaces.

## Additional Information

**How to cite this article**: Zuo, Z.-J. *et al.* Efficient Synthesis of Ethanol from CH_4_ and Syngas on a Cu-Co/TiO_2_ Catalyst Using a Stepwise Reactor. *Sci. Rep.* **6**, 34670; doi: 10.1038/srep34670 (2016).

## Supplementary Material

Supplementary Information

## Figures and Tables

**Table 1:** The STY (mg·g_cat_.^−1^·h^−1^) and selectivity (%) of products on Cu-Co/TiO_2_ catalyst.

	CH_3_OH	C_2_H_5_OH	CH_3_COOH	C_2_H_6_	H_2_O
STY	1.90	139.37	0.51	—c	—
selectivity^a^	1.34	98.30	0.36	—	—
s^b^	11.23	88.77	0	0	0

^a^Experiment result.

^b^Microkinetic modeling.

^c^Not detected by experiment.

**Table 2:** The adsorption energies (E_ads_, eV) and adsorption configurations (d, Å) of possible intermediates at their preferable adsorption sites.

Species	E_ads_	d_Cu-X_ (Å)^a^	d_Co-X_(Å)	Adsorption site
CH_4_	−0.11			—
CH_3_	−1.69		2.214	bri_Co_
CH_2_	−4.23	2.153	2.058	fcc_Co_
CH	−5.81	2.065	1.958	fcc_Co_
C	−6.43		1.924	bri_Co_
H	−2.67	1.874	1.764	fcc_Co_
CO	−1.27		2.030	bri_Co_
CO_2_	−0.53		−1.924	top_Co_
H_2_O	−0.08			—
CHO	−1.76		2.203	bri_Co_
COH	−2.72	2.153	1.971	fcc_Co_
O	−3.94	2.012		fcc_Cu_
CH_2_O	−0.26			—
CHOH	−2.21		2.083	bri_Co_
CH_3_O	−1.92		2.125	bri_Co_
CH_2_OH	−1.43		2.118	top_Co_
CH_3_OH	−0.51		2.467	top_Co_
C_2_H_6_	−0.24			—
CH_3_CO	−1.88		2.052	top_Co_
	−1.82	2.039		top_Cu_
CH_2_CO	−1.67	2.256	2.124	fcc_Co_/C(-H)-bri_Co_,C(-O)-top_Cu_
CHCO	−3.12	2.384	2.037	fcc_Co_/C(-H)-bri_Co_,C(-O)-top_Cu_
CCO	−4.94	2.147	1.979	fcc_Co_
CH_3_COH	−2.34		1.987	top_Co_
CH_3_CHO	−0.59		2.040	top_Co_
CH_3_CHOH	−1.75		2.171	top_Co_
C_2_H_5_OH	−0.43		2.289	top_Co_
CH_3_COO	−0.92		2.073	bri_Co_/O-top_Co_, O-top_Co_
CH_3_COOH	−0.27			—

^a^The nearest bond length, X stands for H, C or O.

**Table 3:** The optimal reaction pathways for ethanol synthesis on CoCu(111) surface together with the corresponding activation barriers(E_a_, eV).

No.	Elementary reactions	E_a_	No.	Elementary reactions	E_a_
1	CH_4_(g) + * → CH_4_*		12	CH_2_* + H* → CH_3_* + *	0.61
2	CO(g) + * → CO*		13	CH_4_* + * → CH_3_* + H*	1.28
3	H_2_(g) + 2* → 2H*		14	CH_3_* + CO* → CH_3_CO* + H*	0.49
4	CO* + H* → CHO* + *	1.09	15	CH_3_CO* + H*→ CH_3_COH * + *	0.86
5	CHO* + H* → CH_2_O* + *	0.72	16	CH_3_COH* + H*→ CH_3_CHOH* + *	0.62
6	CH_2_O* + H* → CH_3_O* + *	0.89	17	CH_3_CHOH* + H*→ C_2_H_5_OH(g) + *	0.28
7	CH_2_O* + H* → CH_2_OH* + *	0.82	18	CO* + O* → CO_2_* + *	0.81
8	CH_3_O* + * → CH_3_* + O*	0.62	19	CH_3_* + CO_2_* → CH_3_COO* + *	1.13
9	CH_3_O* + H* → CH_3_OH(g) + 2*	1.17	20	CH_3_COO* + H* → CH_3_COOH* + *	0.93
10	CH_2_OH* + * → CH_2_* + OH *	1.09	21	H* + OH* → H_2_O (g) + *	1.43
11	CH_2_OH* + H* → CH_3_OH(g) + 2*	1.38	22	CH_3_* + CH_3_* → C_2_H_6_(g) + *	0.89
