# Supplementary material for: Efficient Synthesis of Ethanol from CH4 and Syngas on a Cu-Co/TiO2 Catalyst Using a Stepwise Reactor
Source: Sci Rep. 2016 Oct 3;6:34670. doi: 10.1038/srep34670 (PMC5046147; doi:10.1038/srep34670)
Supplement: Supplementary Information [file srep34670-s1.pdf]

Efficient Synthesis of Ethanol from CH_4 and Syngas on a Cu-Co/TiO_2 Catalyst Using a Stepwise Reactor

Zhi-Jun Zuo[1], Fen Peng[1,2], Wei Huang[1,*]

[1] Key Laboratory of Coal Science and Technology of Ministry of Education and Shanxi Province, Taiyuan University of Technology, Taiyuan 030024, Shanxi, China; [2] Key Laboratory of Renewable Energy and Gas Hydrate, Guangzhou Institute of Energy Conversion, Chinese Academy of Sciences, Guangzhou, China

Microkinetic Modeling

As shown in Table 3, R1, 2 and 3 were assumed in equilibrium. The equilibrium constants of these three reactions were defined as follow: [1-2]

$K= \exp[(-(\Delta E_{ads} - T\Delta S) / k_BT]$

Here ΔE_{ads}, ΔS, k_B and T were the adsorption energy of the adsorbate, the entropy change of the corresponding gas-phase adsorbate which can be obtained from NIST Chemistry WebBook[3], the Boltzmann constant and reaction temperature.

The rate constant for R4 – R22 reactions were estimated according to:

$$k = A\exp(-\frac{E_a}{k_BT}) = \frac{k_BT}{h}\frac{Q_{TS}}{Q_R}\exp(-\frac{E_a}{k_BT})$$

where h, A, and E_a, Q_{TS} and Q_R were the Planck constant, prefactor, activation barrier, the partition functions per unit volume for a TS and an IS [1-2].

For typical surface reactions involving only a high-vibrational-frequency bond breaking/ formation, q^{vib} at the IS is close to q^{vib} at the TS, and then Q_{TS}/Q_R is close to 1. In these cases, the pre-exponential factor A is about 10^{12}–10^{13} s^{-1} at typical temperatures [1]. In the paper, we choose 10^{13} s^{-1} as the pre-exponential factor A.

The site balance of all intermediate species included in the reaction mechanism can be given in terms of coverage(θx, x=surface species) (Equation 1)[1-2]:

$\theta_{CH_4} + \theta_{CO} + \theta_H + \theta_{CHO} + \theta_{CH_2O} + \theta_{CH_3O} + \theta_{CH_2} + \theta_{CH_3} + \theta_{CH_2OH} + \theta_{CH_3CO} + \theta_{CH_3COH} + \theta_{CH_3CHOH} +$

$\theta_{CO_2} + \theta_O + \theta_{CH_3CO_2} + \theta_{OH} + \theta_* = 1$

The coverages of CH_4, CO and H are $\theta_{CH_4} = P_{CH_4}K_1\theta_*$, $\theta_{CO} = P_{CO}K_2\theta_*$ and $\theta_H = P_{H_2}^{1/2}K_3^{1/2}\theta_*$, respectively. Other possible surface species are described according to the steady-state approximation as follow[4], where the rates for the production and the consumption are equal:

1. CHO: $\frac{d\theta_{CHO}}{dt} = k_4\theta_{CO}\theta_H - k_5\theta_{CHO}\theta_H = 0$

$$\theta_{CHO} = \frac{k_4}{k_5}\theta_{CO} = \frac{k_4}{k_5}P_{CO}K_2\theta_* \qquad (4)$$

2. CH_2O: $\frac{d\theta_{CH_2O}}{dt} = k_5\theta_{CHO}\theta_H - k_6\theta_{CH_2O}\theta_H - k_7\theta_{CH_2O}\theta_H = 0$

$$\theta_{CH_2O} = \frac{k_4}{k_6+k_7}P_{CO}K_2\theta_* \qquad (5)$$

3. CH_3O: $\frac{d\theta_{CH_3O}}{dt} = k_6\theta_{CH_2O}\theta_H - k_8\theta_{CH_3O}\theta_* - k_9\theta_{CH_3O}\theta_H = 0$

$$\theta_{CH_3O} = \frac{k_6}{k_8\theta_* + k_9\theta_H}\theta_{CH_2O}\theta_H = \frac{k_4k_6P_{CO}K_2K_3^{1/2}P_{H_2}^{1/2}}{\left(k_8+k_9K_3^{1/2}P_{H_2}^{1/2}\right)(k_6+k_7)}\theta_*$$

4. CH_2OH: $\frac{d\theta_{CH_2OH}}{dt} = k_7\theta_{CH_2O}\theta_H - k_{10}\theta_{CH_2OH}\theta_* - k_{11}\theta_{CH_2OH}\theta_H = 0$

$$\theta_{CH_2OH} = \frac{k_7}{k_{10}\theta_* + k_{11}\theta_H}\theta_{CH_2O}\theta_H = \frac{k_4k_7P_{CO}K_2K_3^{1/2}P_{H_2}^{1/2}}{\left(k_{10}+k_{11}K_3^{1/2}P_{H_2}^{1/2}\right)(k_6+k_7)}\theta_*$$

5. CH_2: $\frac{d\theta_{CH_2}}{dt} = k_{10}\theta_{CH_2OH}\theta_* - k_{12}\theta_{CH_2}\theta_H = 0$

$$\theta_{CH_2} = \frac{k_{10}\theta_{CH_2OH}\theta_*}{k_{12}\theta_H} = \frac{k_4k_7k_{10}P_{CO}K_2}{\left(k_{10}+k_{11}K_3^{1/2}P_{H_2}^{1/2}\right)(k_6+k_7)k_{12}}\theta_*$$

6. CH_3: $\frac{d\theta_{CH_3}}{dt} = k_{13}\theta_{CH_4}\theta_* + k_8\theta_{CH_3O}\theta_* + k_{12}\theta_{CH_2}\theta_H - k_{14}\theta_{CH_3}\theta_{CO} - k_{19}\theta_{CH_3}\theta_{CO_2} = 0$

$$\theta_{CH_3} = \frac{k_{13}P_{CH_4}K_1\left(k_{10}+k_{11}K_3^{1/2}P_{H_2}^{1/2}\right)(k_6+k_7) + k_4k_7k_{10}K_2P_{CO}K_3^{1/2}P_{H_2}^{1/2}}{\left(k_{10}+k_{11}K_3^{1/2}P_{H_2}^{1/2}\right)(k_6+k_7)k_{14}K_2P_{CO}}\theta_*$$

7. CH_3CO: $\frac{d\theta_{CH_3CO}}{dt} = k_{14}\theta_{CH_3}\theta_{CO} - k_{15}\theta_{CH_3CO}\theta_H = 0$

$$\theta_{CH_3CO} = \frac{k_{14}\theta_{CH_3}\theta_{CO}}{k_{15}\theta_H} = \frac{k_{13}P_{CH_4}K_1\left(k_{10}+k_{11}K_3^{1/2}p_{H_2}^{1/2}\right)(k_6+k_7)+k_4k_7k_{10}K_2\,P_{CO}\,K_3^{1/2}p_{H_2}^{1/2}}{k_{15}K_3^{1/2}p_{H_2}^{1/2}\left(k_{10}+k_{11}K_3^{1/2}p_{H_2}^{1/2}\right)(k_6+k_7)}\theta_*$$

8. CH_3COH: $\frac{d\theta_{CH_3COH}}{dt} = k_{15}\theta_{CH_3CO}\theta_H - k_{16}\theta_{CH_3COH}\theta_H = 0$

$$\theta_{CH_3COH} = \frac{k_{15}\theta_{CH_3CO}}{k_{16}} = \frac{k_{13}P_{CH_4}K_1\left(k_{10}+k_{11}K_3^{1/2}p_{H_2}^{1/2}\right)(k_6+k_7)+k_4k_7k_{10}K_2\,P_{CO}\,K_3^{1/2}p_{H_2}^{1/2}}{k_{16}K_3^{1/2}p_{H_2}^{1/2}\left(k_{10}+k_{11}K_3^{1/2}p_{H_2}^{1/2}\right)(k_6+k_7)}\theta_*$$

9. CH_3CHOH: $\frac{d\theta_{CH_3CHOH}}{dt} = k_{16}\theta_{CH_3COH}\theta_H - k_{17}\theta_{CH_3CHOH}\theta_H = 0$

$$\theta_{CH_3CHOH} = \frac{k_{16}\theta_{CH_3COH}}{k_{17}} = \frac{k_{13}P_{CH_4}K_1\left(k_{10}+k_{11}K_3^{1/2}p_{H_2}^{1/2}\right)(k_6+k_7)+k_4k_7k_{10}K_2\,P_{CO}\,K_3^{1/2}p_{H_2}^{1/2}}{k_{17}K_3^{1/2}p_{H_2}^{1/2}\left(k_{10}+k_{11}K_3^{1/2}p_{H_2}^{1/2}\right)(k_6+k_7)}\theta_*$$

10. CO_2: $\frac{d\theta_{CO_2}}{dt} = k_{18}\theta_O\theta_{CO} - k_{19}\theta_{CH_3}\theta_{CO_2} = 0$

$$\theta_{CO_2} = \frac{k_{18}\theta_O\theta_{CO}}{k_{19}\theta_{CH_3}} = \frac{\left(k_{10}+k_{11}K_3^{1/2}p_{H_2}^{1/2}\right)k_4k_6k_8k_{14}K_3^{1/2}p_{H_2}^{1/2}\,K_2^2P_{CO}^2}{\left(k_8+k_9K_3^{1/2}p_{H_2}^{1/2}\right)k_{19}\,\{k_{13}P_{CH_4}K_1\left(k_{10}+k_{11}K_3^{1/2}p_{H_2}^{1/2}\right)(k_6+k_7)+k_4k_7k_{10}K_2\,P_{CO}\,K_3^{1/2}p_{H_2}^{1/2}\,\}}\theta_*$$

11. O: $\frac{d\theta_O}{dt} = k_8\theta_{CH_3O}\theta_* - k_{18}\theta_O\theta_{CO} = 0$

$$\theta_O = \frac{k_8\theta_{CH_3O}\theta_*}{k_{18}\theta_{CO}} = \frac{k_4k_6k_8K_3^{1/2}p_{H_2}^{1/2}}{\left(k_8+k_9K_3^{1/2}p_{H_2}^{1/2}\right)(k_6+k_7)k_{18}}\theta_*$$

12. CH_3CO_2: $\frac{d\theta_{CH_3CO_2}}{dt} = k_{19}\theta_{CH_3}\theta_{CO_2} - k_{20}\theta_{CH_3CO_2}\theta_H = 0$

$$\theta_{CH_3CO_2} = \frac{k_{19}\theta_{CH_3}\theta_{CO_2}}{k_{20}\theta_H} = \frac{k_4k_6k_8K_2\,P_{CO}}{\left(k_8+k_9K_3^{1/2}p_{H_2}^{1/2}\right)(k_6+k_7)k_{20}}\theta_*$$

13. OH : $\frac{d\theta_O}{dt} = k_{10}\theta_{CH_2OH}\theta_* - k_{21}\theta_H\theta_{OH} = 0$

$$\theta_{OH} = \frac{k_{10}\theta_{CH_2OH}\theta_*}{k_{21}\theta_H} = \frac{k_4k_7k_{10}P_{CO}K_2}{k_{21}\left(k_{10}+k_{11}K_3^{1/2}p_{H_2}^{1/2}\right)(k_6+k_7)}\theta_*$$

Therefore,

$$P_{CH_4}K_1\theta_* + P_{CO}K_2\theta_* + P_{H_2}^{1/2}K_3^{1/2}\theta_* + \frac{k_{13}P_{CH_4}K_1\left(k_{10}+k_{11}K_3^{1/2}p_{H_2}^{1/2}\right)(k_6+k_7)+k_4k_7k_{10}K_2\,P_{CO}\,K_3^{1/2}p_{H_2}^{1/2}}{k_{15}K_3^{1/2}p_{H_2}^{1/2}\left(k_{10}+k_{11}K_3^{1/2}p_{H_2}^{1/2}\right)(k_6+k_7)}\theta_*$$

$$+ \frac{\left(k_{10}+k_{11}K_3^{1/2}p_{H_2}^{1/2}\right)k_4k_6k_8k_{14}K_3^{1/2}p_{H_2}^{1/2}\,K_2^2P_{CO}^2}{\left(k_8+k_9K_3^{1/2}p_{H_2}^{1/2}\right)k_{19}\,\{k_{13}P_{CH_4}K_1\left(k_{10}+k_{11}K_3^{1/2}p_{H_2}^{1/2}\right)(k_6+k_7)+k_4k_7k_{10}K_2\,P_{CO}K_3^{1/2}p_{H_2}^{1/2}\,\}}\theta_* + \frac{k_4}{k_5}P_{CO}K_2\theta_*$$

$$+ \frac{k_{13}P_{CH_4}K_1\left(k_{10}+k_{11}K_3^{1/2}p_{H_2}^{1/2}\right)(k_6+k_7)+k_4k_7k_{10}K_2\,P_{CO}\,K_3^{1/2}p_{H_2}^{1/2}}{\left(k_{10}+k_{11}K_3^{1/2}p_{H_2}^{1/2}\right)(k_6+k_7)k_{14}K_2\,P_{CO}}\theta_* + \frac{k_4k_7k_{10}P_{CO}K_2}{\left(k_{10}+k_{11}K_3^{1/2}p_{H_2}^{1/2}\right)(k_6+k_7)k_{12}}\theta_* +$$

$$\frac{k_4k_6P_{CO}K_2\,K_3^{1/2}p_{H_2}^{1/2}}{\left(k_8+k_9K_3^{1/2}p_{H_2}^{1/2}\right)(k_6+k_7)}\theta_* + \frac{k_{13}P_{CH_4}K_1\left(k_{10}+k_{11}K_3^{1/2}p_{H_2}^{1/2}\right)(k_6+k_7)+k_4k_7k_{10}K_2\,P_{CO}\,K_3^{1/2}p_{H_2}^{1/2}}{k_{16}K_3^{1/2}p_{H_2}^{1/2}\left(k_{10}+k_{11}K_3^{1/2}p_{H_2}^{1/2}\right)(k_6+k_7)}\theta_* +$$

$$\frac{k_4k_7P_{CO}K_2K_3^{1/2}p_{H_2}^{1/2}}{\left(k_{10}+k_{11}K_3^{1/2}p_{H_2}^{1/2}\right)(k_6+k_7)}\theta_* + \frac{k_{13}P_{CH_4}K_1\left(k_{10}+k_{11}K_3^{1/2}p_{H_2}^{1/2}\right)(k_6+k_7)+k_4k_7k_{10}K_2\,P_{CO}\,K_3^{1/2}p_{H_2}^{1/2}}{k_{17}K_3^{1/2}p_{H_2}^{1/2}\left(k_{10}+k_{11}K_3^{1/2}p_{H_2}^{1/2}\right)(k_6+k_7)}\theta_*$$

$$+\frac{k_4}{k_6+k_7}P_{CO}K_2\theta_* + \frac{k_4k_6k_8K_3^{1/2}p_{H_2}^{1/2}}{\left(k_8+k_9K_3^{1/2}p_{H_2}^{1/2}\right)(k_6+k_7)k_{18}}\theta_* + \frac{k_4k_6k_8K_2\,P_{CO}}{\left(k_8+k_9K_3^{1/2}p_{H_2}^{1/2}\right)(k_6+k_7)k_{20}}\theta_* +$$

$$\frac{k_4k_7k_{10}P_{CO}K_2}{k_{21}\left(k_{10}+k_{11}K_3^{1/2}p_{H_2}^{1/2}\right)(k_6+k_7)}\theta_* + \theta_* = 1 \quad \text{(Equation 2)}$$

It should be pointed out the R22 reaction is not included in the Equation 2. If R22 is considered the Equation 2, the Equation is a quadratic equation with one unknown. The question is very hard to obtain. Therefore, R22 is not considered in the Equation 2

The relative reaction ratio of CH_3OH, C_2H_5OH, C_2H_6, CH_3COOH and H_2O are $r_{CH_3OH} = k_9\theta_{CH_3O}\theta_H + k_{11}\theta_{CH_2OH}\theta_H$, $r_{C_2H_5OH} = k_{17}\theta_{CH_3CHOH}\theta_H$, $r_{CH_3CH_3} = k_{22}\theta_{CH_3}\theta_{CH_3}$, $r_{CH_3COOH} = k_{20}\theta_{CH_3CO_2}\theta_H$ and $R_{H_2O} = k_{21}\theta_{OH}\theta_H$.

The relative selectivity (s) are defined as: $s_i = r_i/\sum_i r_i$, where r is relative rate for each product, i is CH_3OH, C_2H_5OH, C_2H_6, CH_3COOH and H_2O.

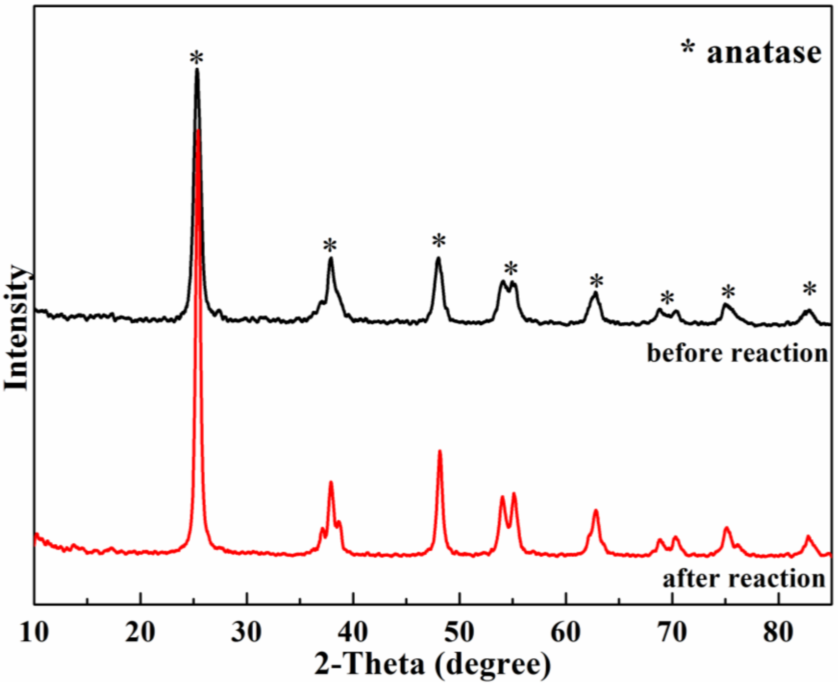

Fig. S1 XRD pattern of the Cu-Co/TiO_2 catalyst before and after reaction

Fig. S1 shows the X-ray powder diffraction (XRD) of the Cu-Co/TiO_2 catalyst before and after the reaction. Only anatase peaks are found before and after the reaction[5-6], and no other element is found. This result indicates that the Cu species and Co species are uniformly dispersed on the catalyst surface, which is in agreement with the high-resolution transmission electron microscopy result (Fig. S2); the particle size is determined to be approximately 125 Å. After reaction, no new peak appears, indicating that the phase transition from anatase to rutile does not occur at 500 ℃. This observation is in agreement with previous studies, in which the phase transition occurs over a wide range of temperatures above 600 ℃[7-8]. However, the peak intensity increases after reaction, indicating that the particle size increases.

The catalyst was determined from XRD patterns collected on a RigakuD/max-2500 diffractometer with Cu Kα radiation (40 kV/100 mA) at 8 °/min scanning rate in the range of 10–85 °.

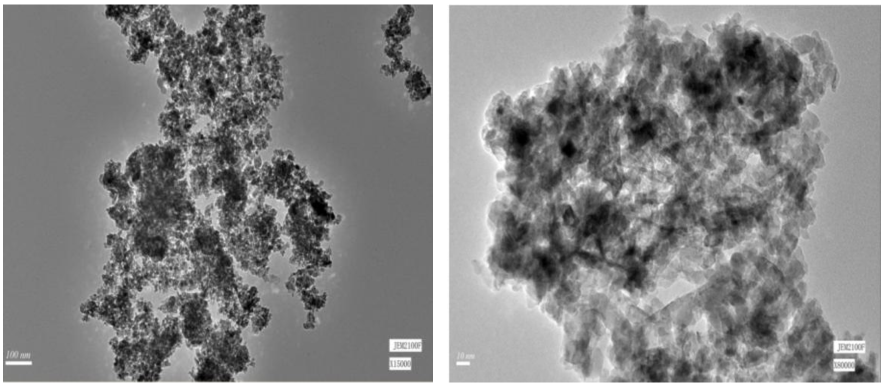

Fig. S2 The TEM image before reaction

The morphology was studied using the high-resolution transmission electron microscopy (JEM-2100F). It was found that Co and Cu species were uniformly dispersed on the catalyst surface, and the particle size is about 125 Å.

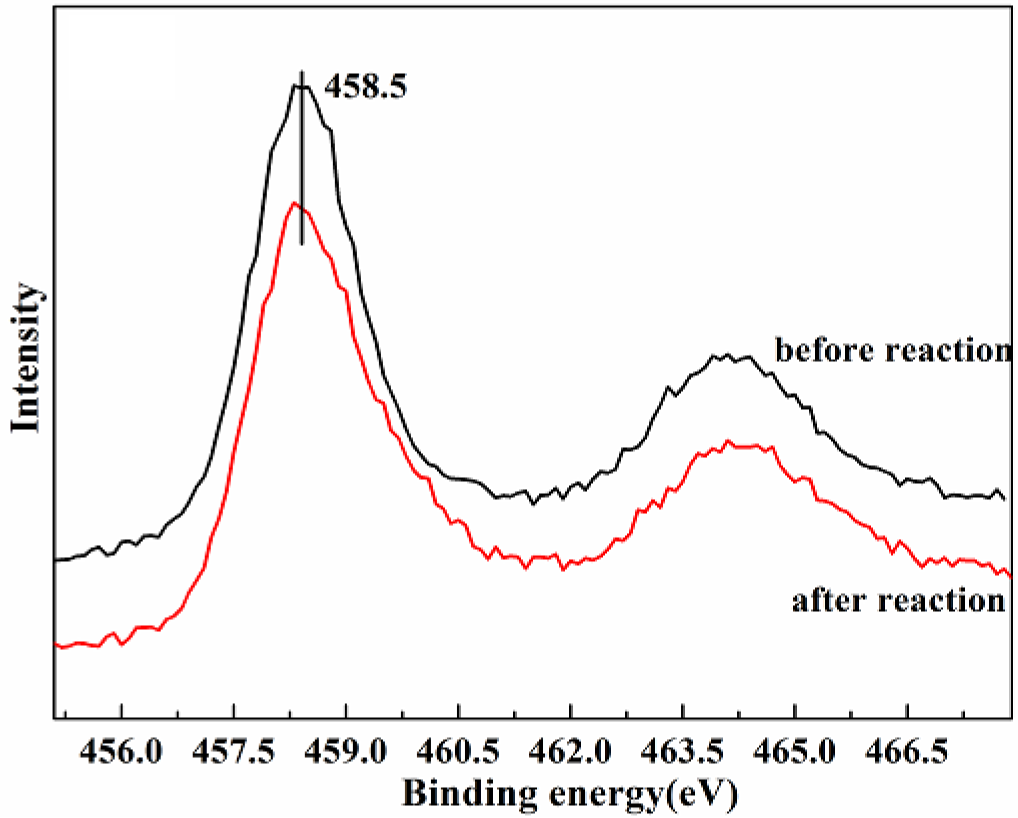

Fig. S3 Ti 2p XPS spectra before and after reaction

The Calculation Model

Previous studies found that Cu oxidation was easily reduced and that CoO was the primary phase under 400 ℃ using H_2 reduction[6,9-11]. Therefore, Cu and CoO were the primary phases in the Cu-Co/TiO_2 catalyst; our XPS analysis confirmed the result (see the XPS section). We proposed that ethanol synthesis from CH_4 and syngas requires two active sites of Cu and CoO. Therefore, the interface of CoO and Cu was suitable for our catalyst. However, the main difficulty encountered in the work is the lack of information regarding the geometrical structure of the particular Cu−CoO interface. Therefore, a CuCo alloy represented the Cu-CoO interface in the paper, and we think this model can reflect the reaction of ethanol synthesis from CH_4 and syngas to a certain extent. Recently, various types of alloys have been used and studied for different reactions by many researchers[12-19]. For example, the Chen group studied the methanol decomposition on a PdZn alloy using DFT. They found that the energy barrier of CH_3O dehydrogenation on a PdZn(111) surface was higher than that on a Pd(111) surface because the binding strength of CH_3O on the Pd(111) surface is weaker than that on the PdZn(111) surface. Their results were in agreement with the experiment result[16,19].

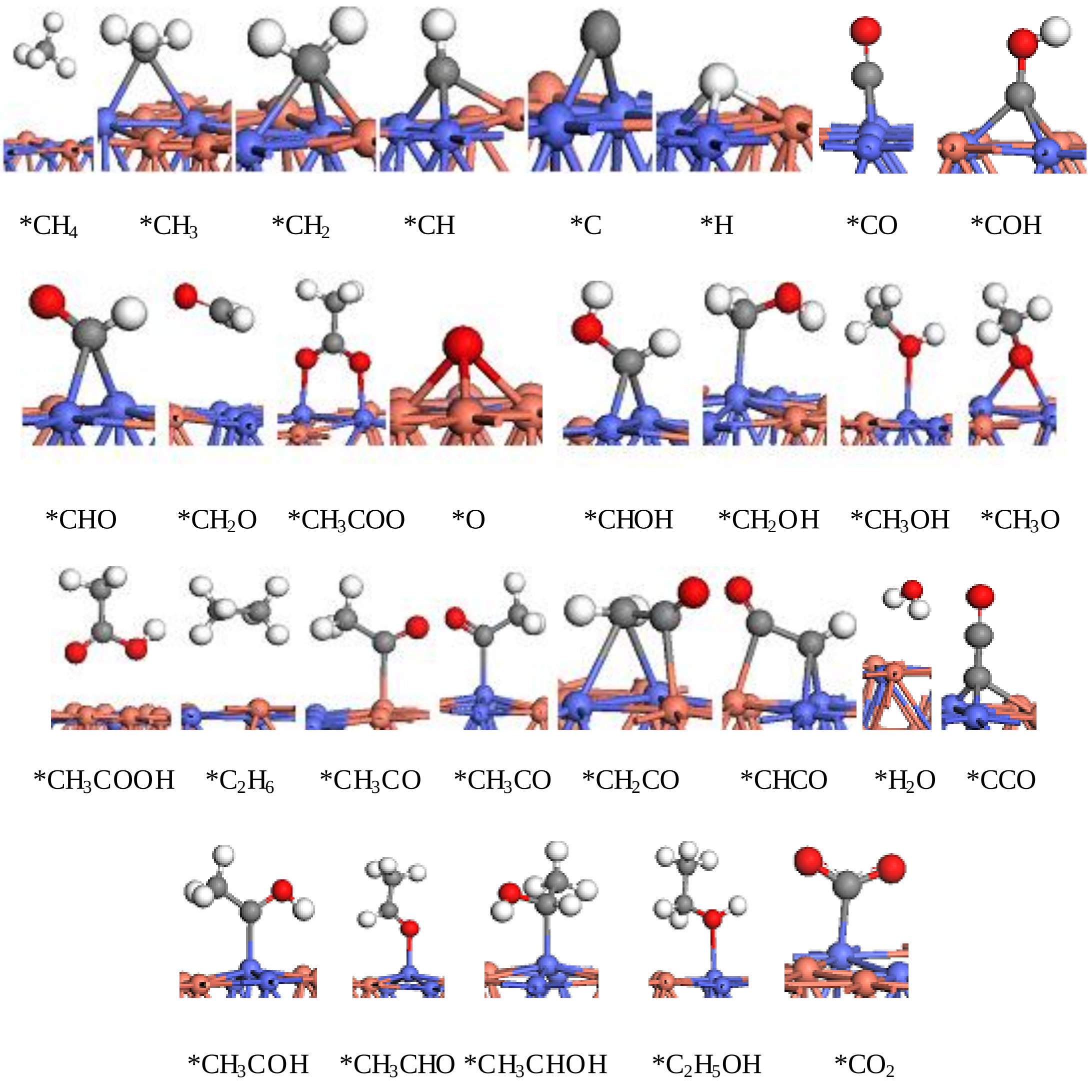

Fig. S4 The most stable adsorption configuration of possible intermediates adsorption on CoCu(111) surface during ethanol synthesis from CH_4 and syngas

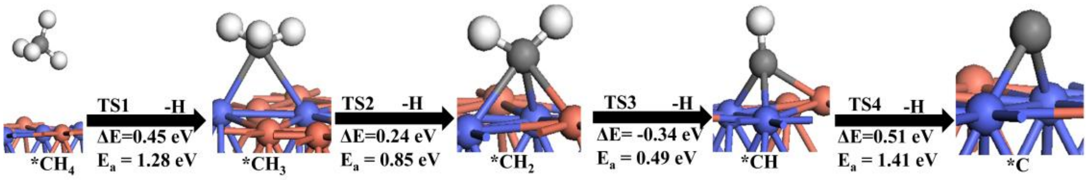

Fig. S5 Energy barriers (E_a, eV) and reaction energies (ΔE, eV) of *CH_4 dehydrogenation to *C on the CoCu(111) surface

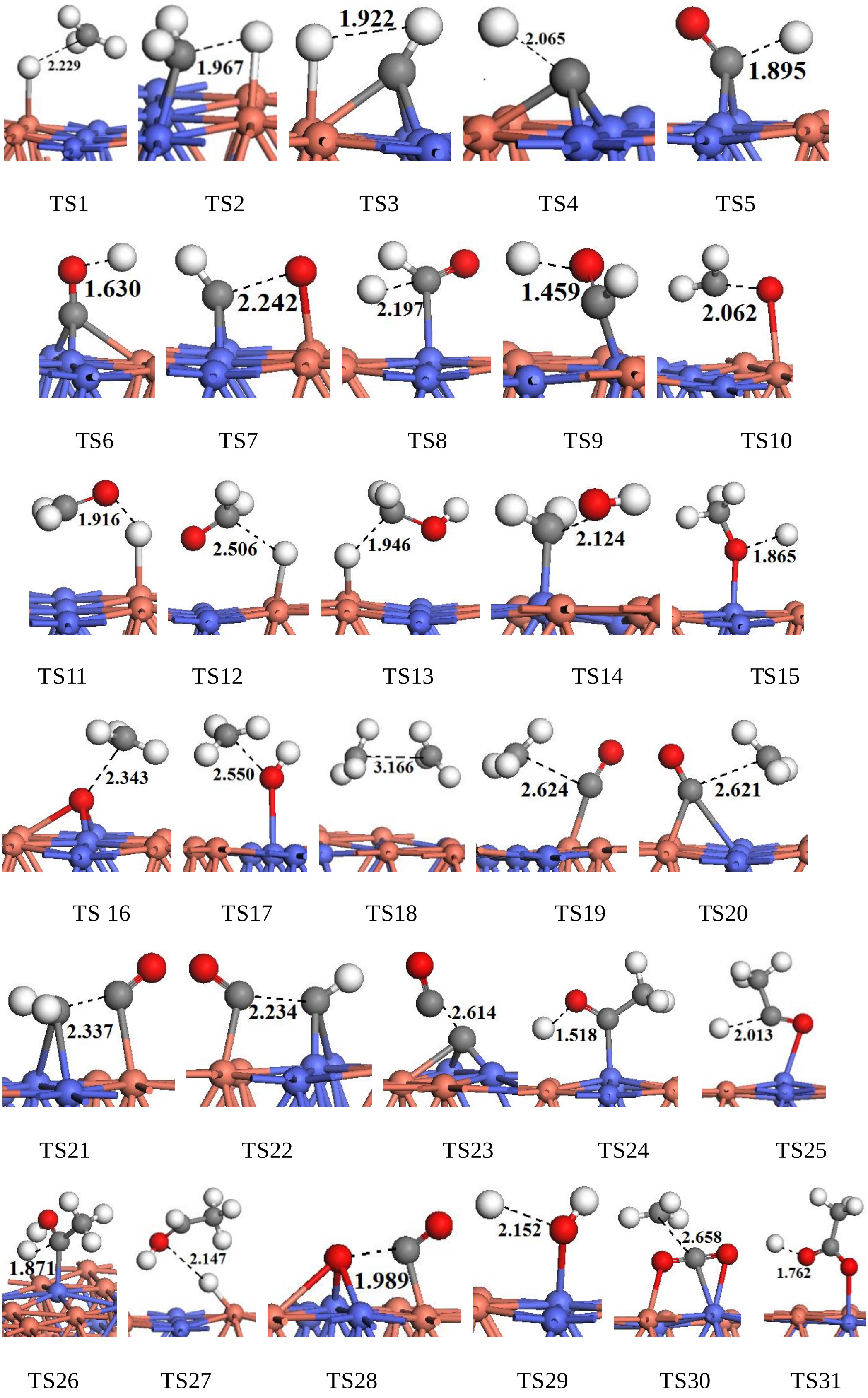

Fig. S6 The TS structure during ethanol synthesis from CH_4-syngas on CoCu(111) surface

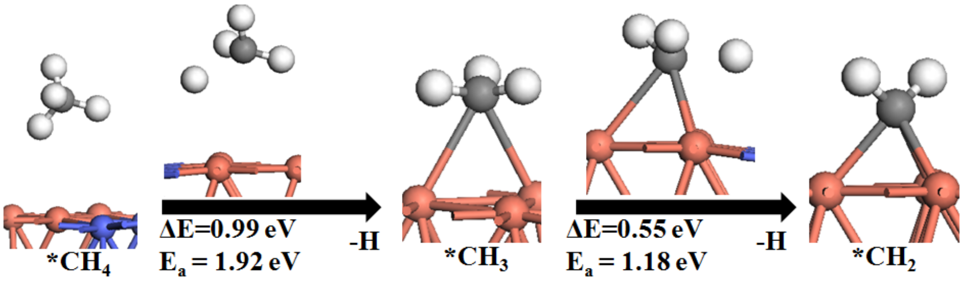

Fig. S7 the energy barriers, reaction energies and TS structures of *CH_4 dehydrogenation to *CH_2 on CoCu(111) surface

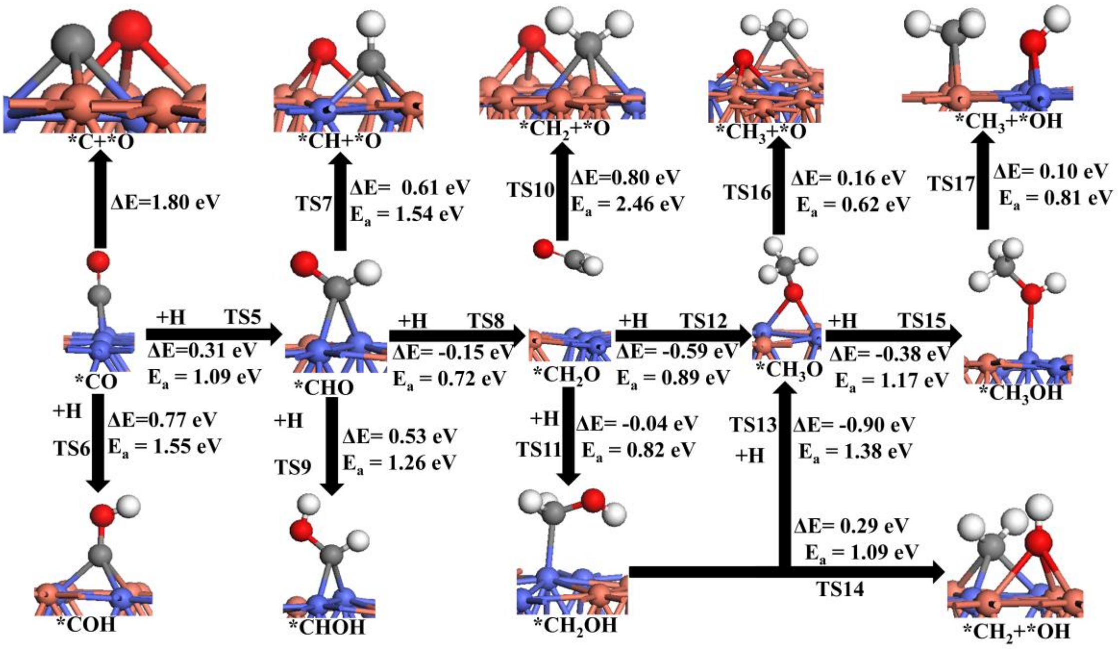

Fig. S8 Energy barriers (E_a, eV) and reaction energies (ΔE, eV) of *CO hydrogenation to *CH_3OH on the CoCu(111) surface

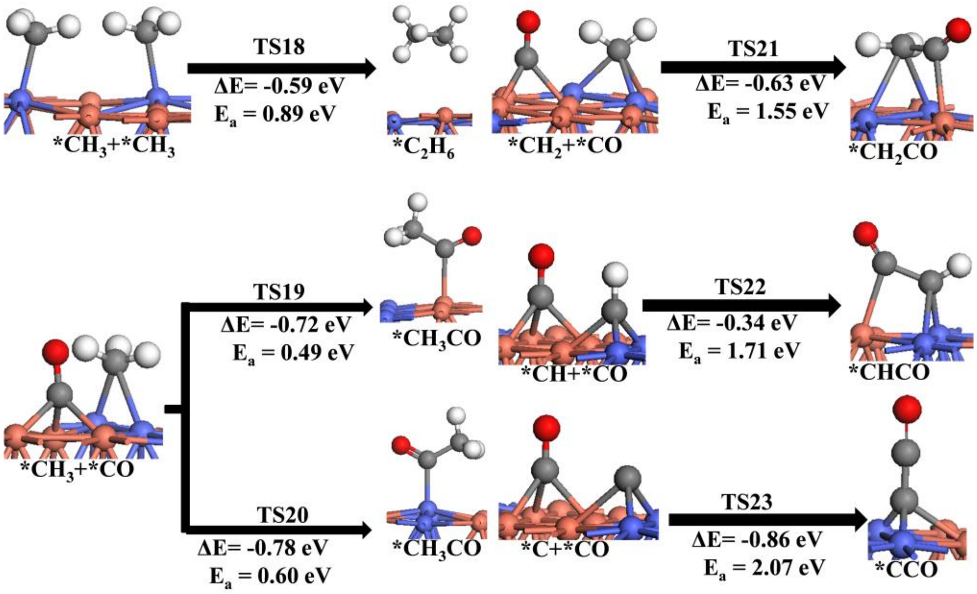

Fig. S9 Energy barriers (E_a, eV) and reaction energies (ΔE, eV) of the C-C formation from *CO reaction with $*CH_3$, *CO, $*CH_2$, *CH and *C on the CoCu(111) surface

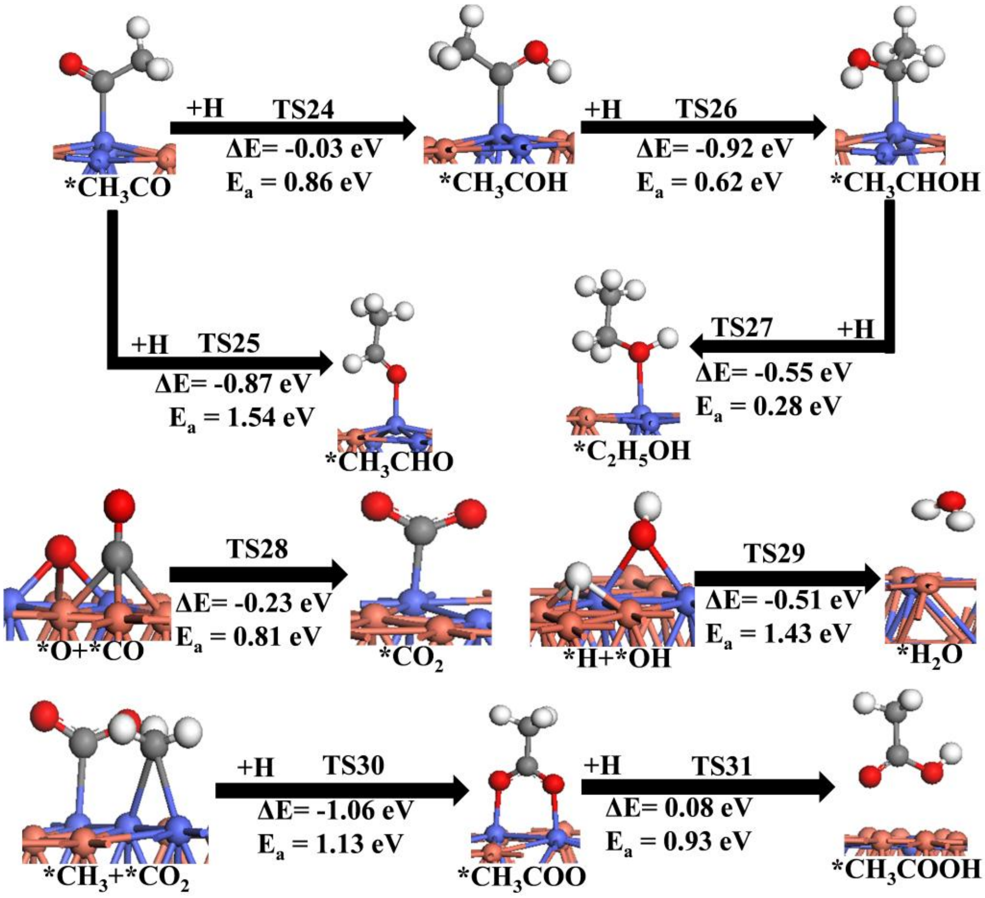

Fig. S10 Energy barriers (E_a, eV) and reaction energies (ΔE, eV) of *C_2H_5OH formation from *CH_3CO hydrogenation, *CO_2, *H_2O and *CH_3COOH formation from *CH_3 reaction with *CO_2 on the CoCu(111) surface

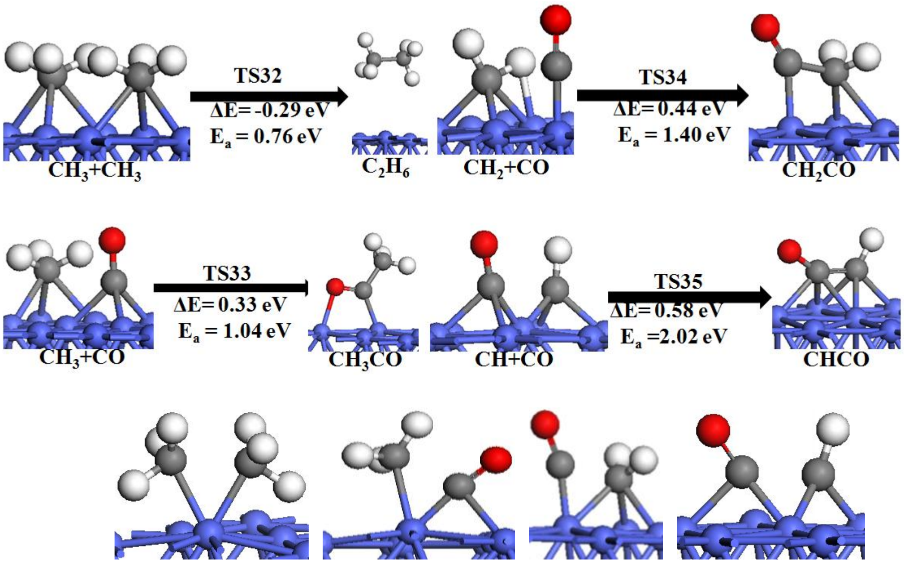

Fig. S11 the energy barriers, reaction energies and TS structures of $*C_2H_6$, $*CH_3CO$, $*CH_2CO$ and *CHCO formation on the Co(111) surface

Table S1 the adsorption energies (E_{ads}, eV) and adsorption configurations of possible intermediates at Cu sites.

	E_{ads}	d_{Cu-X}[a] (Å)	Adsorption site
CH_4	-0.10		
CH_3	-1.23	2.395	fcc_{Cu}
CH_2	-3.82	2.177	fcc_{Cu}
CH	-5.21	2.000	fcc_{Cu}
C	-5.46	2.013	fcc_{Cu}
H	-2.45	1.806	3Cu

[a] the nearest bond length, X stands for H or C

1. Tang, Q.-L. Hong, Q.-J. & Liu, Z.-P. Co_2 Fixation into Methanol at Cu/ZrO_2 Interface from First Principles Kinetic Monte Carlo. *J. Catal.* **263**, 114-122, (2009).

2. Choi, Y. & Liu, P. Mechanism of Ethanol Synthesis from Syngas on Rh(111). *J. Am. Chem. Soc.* **131,** 13054-13061, (2009).

3. http://webbook.nist.gov/chemistry/.

4. Liu, P. Logadottir, A. & Nørskov, J. K. Modeling the Electro-Oxidation of Co and H_2/CO on Pt, Ru, Ptru and Pt_3Sn. *Electr. Acta* **48**, 3731-3742, (2003).

5. Shirke, B. S., Korake, P. V., Hankare, P. P., Bamane, S. R. & Garadkar, K. M. Synthesis and Characterization of Pure Anatase TiO_2 Nanoparticles. *J. Mater. Sci.* **22**, 821-824 (2011).

6. Huang, W., Zuo, Z., Han, P., Li, Z. & Zhao, T. XPS and XRD investigation of Co/Pd/TiO_2 catalysts by different preparation methods. *J. Electron. Spectrosc. Relat. Phenom.* **173**, 88-95,

(2009).

7. Mazaheri, M., Razavi H. Z. & Sadrnezhaad, S. K. Two-Step Sintering of Titania Nanoceramics Assisted by Anatase-to-Rutile Phase Transformation. *Scri. Mater.* **59,** 139-142 (2008).

8. Hague, D. C. & Mayo, M. J. Controlling Crystallinity During Processing of Nanocrystalline Titania. *J. Ame. Cera. Soc.*, **77**, 1957-1960, (1994).

9. Zuo, Z. J., Wang, L., Liu, Y. J. & Huang, W. The effect of CuO–ZnO–Al_2O_3 catalyst structure onthe ethanol synthesis from syngas. *Catal. Commun.* **34**, 69-72, (2013).

10. Jeong, Y. et al. Alcohol-assisted low temperature methanol synthesis from syngas over Cu/ZnO catalysts: Effect of pH value in the co-precipitation step. *J. Mol. Catal. A* **400**, 132-138, (2015).

11. Papavasiliou, J., Avgouropoulos, G. & Ioannides, T. Effect of dopants on the performance of CuO–CeO_2 catalysts in methanol steam reforming. *Appl. Catal. B* **69**, 226-234, (2007).

12. Zhang, N. et al. Origins for the synergetic effects of $AuCu_3$ in catalysis for oxygen reduction reaction. *J. Phys. Chem. C* **119**, 907-912, (2015).

13. Wang, X., Chen, L. & Li, B. A density functional theory study of methanol dehydrogenation on the $PtPd_3$(111) surface. *Int. J. Hydrogen Energy* **40**, 9656-9669, (2015).

14. Krajčí, M., Tsai, A. P. & Hafner, J. Understanding the selectivity of methanol steam reforming on the (111) surfaces of NiZn, PdZn and PtZn: Insights from DFT. *J. Catal.* **330**, 6-18, (2015).

15. Liu, P., Yang, Y. & White, M. G. Theoretical perspective of alcohol decomposition and synthesis from CO_2 hydrogenation. *Surf. Sci. Rep.* **68**, 233-272, (2013).

16. Huang, Y., He, X. & Chen, Z.-X. Density functional study of methanol decomposition on clean and O or OH adsorbed PdZn(111). *J. Chem. Phys.* **138**, 184701, (2013).

17 Studt, F. et al. CO hydrogenation to methanol on Cu–Ni catalysts: Theory and experiment. *J. Catal*. **293**, 51-60, (2012).

18. Gan, L.Y., Tian, R.Y., Yang, X.B., Lu, H.D. & Zhao, Y.J. Catalytic reactivity of CuNi alloys toward H_2O and CO dissociation for an efficient water–gas shift: A DFT study. *J. Phys. Chem. C* **116**, 745-752, (2012).

19. Chen, Z. X. Neyman, K. M., Lim, K. H. & Rösch, N. CH_3O decomposition on PdZn(111), Pd(111), and Cu(111). A theoretical study. *Langmuir* **20**, 8068-8077, (2004).
